# Comprehensive Geriatric Health Assessment Core Competencies and Skills for Primary Care Nurses: A Scoping Review

**DOI:** 10.3390/geriatrics10020048

**Published:** 2025-03-18

**Authors:** Ioanna Dimitriadou, Eloranta Sini, Jekaterina Šteinmiller, Maria Saridi, Anna Lundberg, Magdalena Häger, Ingibjorg Hjaltadottir, Sigrun S. Skuladottir, Nina Korsström, Susanna Mört, Hannele Tuori, Evangelos C. Fradelos

**Affiliations:** 1 Laboratory of Clinical Nursing, Department of Nursing, University of Thessaly, 41222 Larissa, Greece; saridim@hotmail.com (M.S.); evagelosfradelos@hotmail.com (E.C.F.); 2 Faculty of Health and Wellbeing, Turku University of Applied Sciences, 20520 Turku, Finland; sini.eloranta@turkuamk.fi (E.S.); susanna.mort@turkuamk.fi (S.M.); 3 Department of Nursing Science, University of Turku, 20014 Turku, Finland; 4 Department of Nursing, Tallinn Health Care College, 13418 Tallinn, Estonia; jekaterina.steinmiller@ttk.ee; 5 Department of Nursing, Åland University of Applied Sciences, 22100 Mariehamn, Finland; annasofia.lundberg@ha.ax (A.L.); magdalena.hager@ha.ax (M.H.); 6 Faculty of Nursing and Midwifery, School of Health Sciences, University of Iceland, 101 Reykjavík, Iceland; ingihj@hi.is (I.H.); sigrunsunna@hi.is (S.S.S.); 7 Department of Emergency Care, Public Health and Midwifery, Turku University of Applied Sciences, 20520 Turku, Finland; nina.korsstrom@turkuamk.fi; 8 The Wellbeing Services County of Southwest Finland, Turku University Hospital, Domain of General Practice and Rehabilitation, 20521 Turku, Finland; hannelt@student.uef.fi

**Keywords:** comprehensive geriatric assessment, primary care nurses, nursing competencies, geriatric care, patient-centered care

## Abstract

**Objective:** This scoping review aims to explore and synthesize the core competencies and skills required for primary care nurses conducting comprehensive geriatric assessments. Comprehensive geriatric assessments have become integral to providing holistic, patient-centered care for older adults with complex health needs, but the specific competencies required in primary care remain underresearched. **Design:** The review followed Arksey and O’Malley’s five-stage scoping review framework, incorporating studies from PubMed, CINAHL, EMBASE, and the Cochrane Library. A comprehensive search was conducted from May 2014 to May 2024, and a population–concept–context (PCC) framework was used to identify relevant studies. **Results:** Nineteen studies met the inclusion criteria, revealing six key competency domains for nurses involved in comprehensive geriatric assessments: Clinical Assessment and Diagnostic Competencies, Care Planning and Coordination, Professional and Interpersonal Competencies, Environmental and Systemic Competencies, Technical and Procedural Competencies, and Quality Improvement and Evidence-Based Practice. These competencies are essential for providing high-quality care to older adults and supporting integrated, multidisciplinary approaches to geriatric care. **Conclusions:** The identified competency domains provide a structured framework that can enhance primary care nurses’ ability to deliver more effective, individualized, and coordinated care to older adults. However, the standardization of these competencies remains crucial for ensuring consistency in practice.

## 1. Introduction

The aging global population presents significant challenges to healthcare systems, particularly in the management and care of older adults with complex health and social needs. Comprehensive geriatric assessment (CGA) has emerged as a cornerstone in the provision of high-quality, patient-centered care for this demographic [1,2]. CGA is a multidimensional, multidisciplinary process used to evaluate an older person’s medical, psychological, and functional abilities, with the goal of creating a coordinated and integrated health plan and follow-up [3]. A holistic and individualized approach is the core principle of CGAs, which are usually performed either at home or in in- and outpatient settings, less so in primary care due to limited resources [4,5].

Primary healthcare is the first point of contact for patients seeking healthcare services. According to the World Health Organization (2020), the competencies of primary care nurses include proactively expanding and coordinating care for patients and their families, with the goal of positively influencing their physical, mental, and social well-being [6]. The nursing competencies required for completing CGAs differ because of the different settings and models used. There are 11 competencies previously described by Britten et al. (2018) for primary care nurses for conducting CGAs, including “maximizing health outcomes”, “facilitating transitions in care”, “partnering with family members”, and “facilitating choices within legal and ethical frameworks” [7].

The literature highlights several nursing competencies that improve care for older adults, including evidence-based practice, comprehensive knowledge of health and well-being issues, teamwork, care coordination to ensure continuity of care, and ethical competencies [8,9]. As stated by Tate et al. (2024), relational and cultural competence, along with nurses’ professional values and identifying older adult abuse, are essential geriatric-specific competencies that promote person-centered care for older adults and their families [10].

Nurses, particularly those in primary care settings, play a critical role in conducting CGAs. Their involvement spans multiple phases of the process, starting from initial screenings to thorough evaluations, followed by the development of tailored care plans and the continuous management of these plans. This multidisciplinary approach allows nurses to address the diverse and complex needs of older patients, ensuring more personalized and effective care [11]. Certain specialized nursing roles, such as chronic care management nurses, geriatric nurse practitioners, and community health nurses, are specifically designated to support older adults requiring CGA. These healthcare professionals facilitate the coordination of long-term management strategies, conduct assessments in domiciliary and outpatient settings, and collaborate with multidisciplinary teams to optimize patient outcomes. CGA is cost-effective and beneficial to both healthcare providers and patients [12]. Furthermore, the use of CGAs in primary care settings may help reduce the need for hospitalizations among high-risk older patients [13].

In Europe, there is no universally agreed-upon approach or set of instruments that should be included in a CGA [4], whereas the use of instruments requires competent personnel. In addition, there is limited knowledge that a single model is effective across all levels of healthcare or about the roles of different healthcare professionals in performing a CGA in primary care settings [5,14]. Little is known about primary care nurses’ experiences with conducting CGAs [15]. Therefore, this scoping review aims to systematically explore and synthesize the literature on the core competencies and skills necessary for primary care nurses engaged in comprehensive geriatric assessment.

## 2. Materials and Methods

### 2.1. Study Design

A scoping review design was used to systematically analyze and synthesize literature concerning core competencies and skills essential for primary care nurses engaged in comprehensive geriatric assessment. This approach was chosen because it allows for the comprehensive examination of a diverse range of literature, including both quantitative and qualitative studies, to provide a holistic understanding of the topic. By synthesizing findings from various sources, we aim to offer a comprehensive overview of the knowledge and evidence base related to nursing competencies in geriatric assessment, thus informing clinical practice, education, and future research in this area. The review follows the five-stage approach proposed by Arksey and O’ Malley [16], which includes the identification of the research question, identification of relevant studies, study selection, data analysis, and a summary and reporting of the results.

### 2.2. Literature Search

The formulation of the search strategy relied on a population, concept, and context (PCC) framework (Table 1). The framework aided in devising the primary search terms in accordance with the intended scope of the review. A comprehensive and systematic literature search was conducted to identify relevant studies pertaining to core competencies and skills essential for primary care nurses engaged in comprehensive geriatric assessments. Electronic databases, including PubMed, CINAHL, EMBASE, and the Cochrane Library, were systematically searched from May 2014 to May 2024 via a combination of the following vocabulary terms or Medical Subject Headings definitions and keywords. The search strategy was designed and developed in consultation with a clinical librarian.

Additionally, manual searches were conducted through the reference lists of the retrieved articles and relevant review papers to identify any additional studies missed in the electronic database search. Grey literature sources, such as conference proceedings, dissertations, and governmental reports, were also searched to ensure comprehensiveness. The search strategy aimed to capture a broad spectrum of literature, including both quantitative and qualitative studies, as well as theoretical frameworks, guidelines, and expert opinions relevant to the topic. The search terms were adapted to the specific requirements of each database while ensuring consistency across all searches.

Three independent reviewers conducted the selection of eligible articles and frameworks. Their evaluation was based on the relevance of titles and abstracts, followed by a thorough examination of full-text citations against predefined inclusion criteria. To maintain the integrity of the review process, duplicate publications were meticulously identified and excluded. Additionally, the reference lists of the included articles were scrutinized to identify any potentially overlooked studies.

### 2.3. Inclusion and Exclusion Criteria

The inclusion criteria for this scoping review were as follows: studies involving primary care nurses, including nurse practitioners, registered nurses, licensed practical nurses, nurse clinicians, gerontological nurses, community health nurses, and family nurse practitioners; research focusing on core competencies, educational methods, knowledge, and skills essential for comprehensive geriatric assessment; studies set in primary care environments such as outpatient care, community health centers, home health care, and long-term care facilities; quantitative and qualitative studies, including randomized controlled trials, nonrandomized controlled trials, cross-sectional studies, qualitative research, mixed-methods studies, theoretical frameworks, guidelines, and expert opinions; and articles published in English. The exclusion criteria were studies not addressing core competencies or skills for primary care nurses in comprehensive geriatric assessment; studies involving participants other than primary care nurses; protocols; reviews; low-quality studies as assessed by the Mixed Methods Appraisal (MMAT); articles not available in full text; and studies published in languages other than English. These criteria ensured that the included studies were directly relevant for understanding and enhancing the competencies of primary care nurses in conducting comprehensive geriatric assessments.

### 2.4. Search Outcomes

The search strategy yielded a total of 4567 records. Following the removal of 2151 duplicate records, the titles and abstracts of 2416 studies were subjected to screening. Subsequently, 2203 articles that did not meet the predetermined eligibility criteria were excluded. Consequently, 213 studies remained for full-text assessment. However, the full texts of 45 articles were not accessible, leaving a final set of 168 articles available for detailed review. Ultimately, 19 articles met all the inclusion criteria and were included in the final analysis. The literature search and screening process were conducted following the Preferred Reporting Items for Systematic Reviews and Meta-Analyses (PRISMA) guidelines [17].

### 2.5. Data Evaluation

To reduce the risk of bias, upon completion of the literature search, the included studies underwent rigorous evaluation via the MMAT [18]. The MMAT is versatile and allows for the assessment of various study designs, including qualitative research, quantitative descriptive studies, quantitative randomized controlled trials, and mixed-methods studies. The MMAT facilitated the assessment of methodological quality on the basis of criteria such as the clarity of research questions, the appropriateness of the methodological approach, the integration of qualitative and quantitative data, and the interpretation of findings. In our study, we employed the MMAT, which incorporates four distinct sets of criteria tailored to different study designs: (1) a qualitative set for assessing qualitative studies or components, (2) a quantitative set for randomized controlled trials, (3) a quantitative set for nonrandomized controlled trials and cross-sectional studies, and (4) a mixed-methods set for mixed-methods research, including Delphi surveys. Each article was evaluated and assigned a quality score ranging from 1 to 5. The articles were then categorized based on their scores as low quality (0–1), moderate quality (2–3), or high quality (4–5). For further details, please refer to Supplementary Table S1. All included studies were evaluated independently by three reviewers (EFC, ID, MS) for their relevance to the review objectives, particularly in addressing the core competencies and skills essential for primary care nurses in comprehensive geriatric assessment. Any discrepancies or disagreements in the evaluation process were resolved through discussion and consensus among the review team members.

### 2.6. Data Analysis

Data were extracted from the included studies via a standardized form that recorded key information such as author, title, country, research aim, method, sample size, setting, and quality. The form also details nursing competencies and skills, as well as participant perspectives. To ensure accuracy, a third reviewer validated the data. The research team then discussed and synthesized the findings.

Data analysis involves basic descriptive statistics to quantify the frequency of competencies and content analysis to categorize them. The competencies were coded into broad standard domains, with new subcategories created as needed. Two team members (ECF, MS) independently coded the data to ensure consistency. This systematic approach provides a clear framework for understanding essential nursing competencies in comprehensive geriatric assessment. This scoping review protocol was registered in the Open Science Framework (OSF) on 18 February 2025.

## 3. Results

### 3.1. General Characteristics of the Included Studies

**Figure 1:**
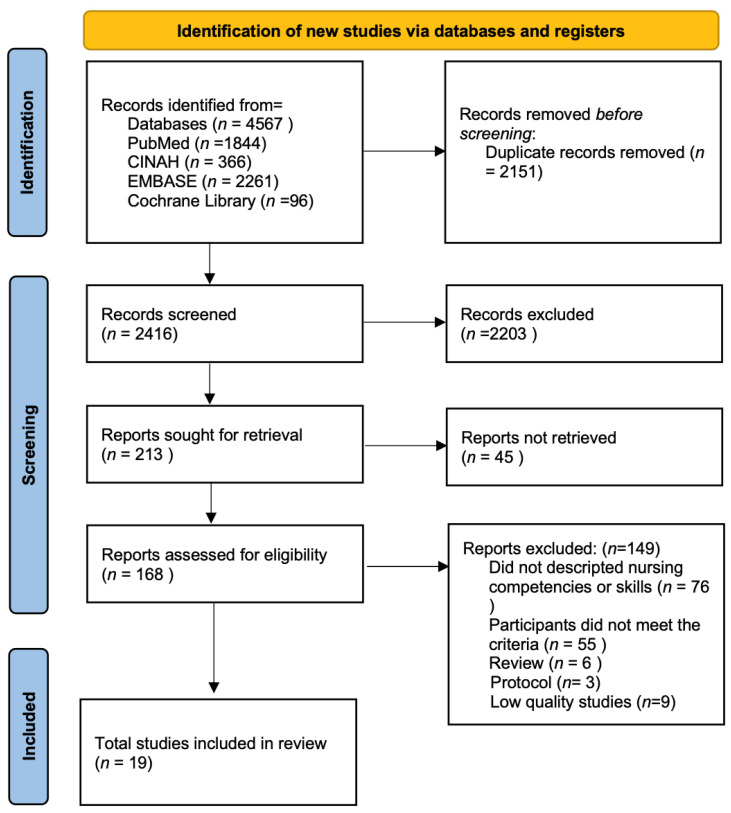
PRISMA flow diagram of study search and selection.

The final review includes 19 studies [8,14,19,20,21,22,23,24,25,26,27,28,29,30,31,32,33,34,35]. The study search and selection process are shown in Figure 1. The final review comprises nineteen studies, including five from the UK; four from Finland; two each from China and Norway; and one each from Canada, Spain, Iran, Sweden, the USA, and Switzerland. The methodological approaches varied: five studies employed the e-Delphi technique, five utilized qualitative methods, four were cross-sectional studies, two were randomized controlled trials (RCTs), one used a mixed-methods approach, one was a quasi-experimental study, and one was an opinion paper. Qualitative studies involved individual interviews, focus groups, and open-ended questionnaires to gather detailed information. The quantitative studies relied on secondary data analysis and structured questionnaires. The mixed-methods studies combined individual interviews and questionnaires to provide a more holistic understanding of the research questions. The general characteristics of the included studies are presented in Table 2.

**Figure 2:**
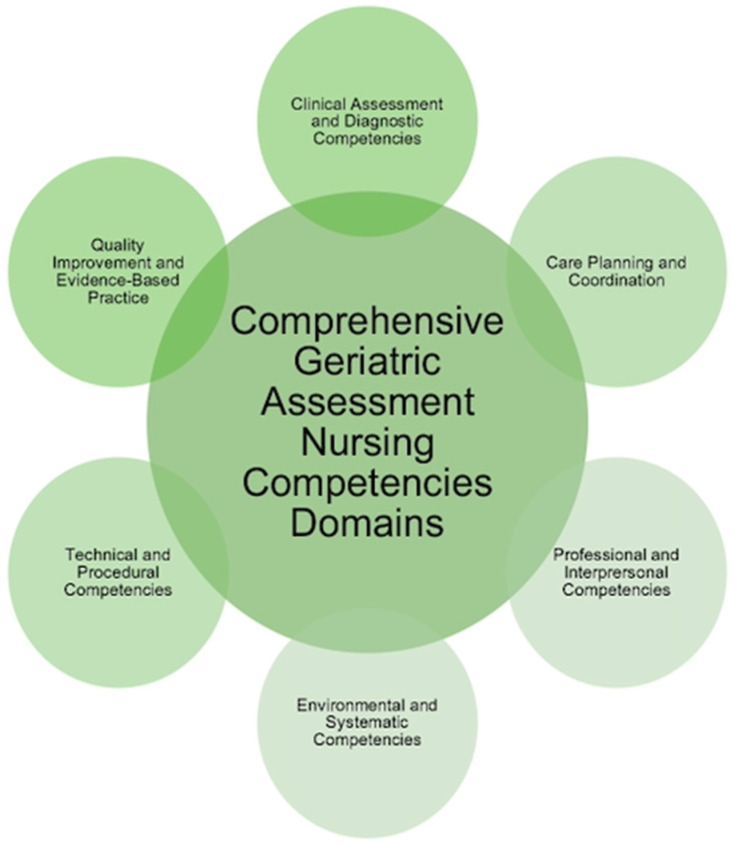
Domains of CGA nursing competencies.

We identified a comprehensive array of competencies and skills essential for primary care nurses conducting comprehensive geriatric assessments. The analysis of the included studies revealed six distinct domains that encapsulate critical areas of competency: Clinical Assessment and Diagnostic Competencies, Care Planning and Coordination, Professional and Interpersonal Competencies, Environmental and Systemic Competencies, Technical and Procedural Competencies, and Quality Improvement and Evidence-Based Practice. Each domain encompasses specific competencies that are integral to providing high-quality, patient-centered care for geriatric older adults (Figure 2).

#### 3.1.1. Clinical Assessment and Diagnostic Competencies

Clinical Assessment and Diagnostic Competencies are foundational to effective geriatric care. The performance of comprehensive health assessments is consistently highlighted across the literature as a critical competency for primary care nurses [19,22,35]. These assessments encompass a wide range of evaluations, including frailty assessment, physical examinations, and the ordering of appropriate diagnostic investigations [20,23,33]. Additionally, the importance of conducting nutritional and cognitive assessments, as well as mood and psychological evaluations, is underscored. Nurses are expected to manage complex clinical scenarios, such as pain management and chronic disease management, which are frequently encountered in geriatric populations [8,19,20,29]. Furthermore, the role of the nurse in specialized assessments, such as medication reviews, delirium management, and the identification of cognitive or physical deficits, is critical to ensuring comprehensive care [23,34,35]. Advanced competencies, including monitoring gait, balance, and fatigue, as well as summarizing clinical findings into actionable problem lists, have also been emphasized [24].

#### 3.1.2. Care Planning and Coordination

A key component of geriatric nursing practice is the creation and management of individualized care plans. Research has shown that nurses must participate in creating customized care plans that are sensitive to the requirements of each patient [14,19,22]. This process is characterized by the active involvement of patients in shared decision-making, which not only respects patient autonomy but also promotes better health outcomes [30]. Maintaining the relevance and efficacy of care plans requires regular reviews and revisions to accommodate evolving patient requirements. Furthermore, a nurse’s responsibility goes beyond providing clinical care to include patient empowerment to control their own health [19,23]. In addition, the literature underscores the importance of end-of-life care planning and the consideration of cultural, spiritual, and emotional needs in the care process. Comprehensive care planning also includes advance care planning (ACP) and palliative care. ACP allows for proactive discussions between patients, families, and healthcare providers about future healthcare decisions, ensuring that care is aligned with the individual’s values and preferences. In addition, palliative care is instrumental in managing symptoms, improving quality of life, and providing psychosocial support to patients with chronic or limiting conditions, regardless of prognosis [33]. The multidimensional character of care planning in geriatric nursing is further demonstrated by competencies linked to environmental assessment, fall prevention, and patient education on safe mobility [23,24].

#### 3.1.3. Professional and Interpersonal Competencies

Lifelong learning and professional development are emphasized as key components of CGAs [27,28,31]. Skills and knowledge, such as healthy aging, geriatric syndromes, and the most common health problems among older people, constitute the cornerstone of CGAs [28].

Multiprofessional cooperation is critical for the effective delivery of CGAs [21,24,26]. In addition, the ability to communicate effectively not only with other professionals but also with older people and their family members is an important part of geriatric assessment [27,28,33]. Building positive relationships with older people and their family members is also essential for delivering care that is respectful and responsive to the diverse needs of older adults [33]. In CGAs, skills related to different cultures are important when providing gerontological nursing care to individuals from different backgrounds [29,33].

#### 3.1.4. Environmental and Systemic Competencies

Competencies in managing the broader environmental and systemic aspects of care are essential for primary care nurses. Creating an open and cooperative work environment is crucial for fostering collaboration and teamwork, which are vital for the delivery of comprehensive geriatric care [32]. Multidisciplinary team collaboration is highlighted as a key competency, with nurses often serving as the linchpin in coordinating care across various healthcare providers [31,34]. Leadership skills, including the management of care teams and advocating for the necessary resources and qualified staff, are critical for maintaining high standards of care [32,33]. Additionally, the ability to independently manage care and contribute to the development of evidence-based practices and policies ensures that primary care nurses can adapt to the evolving needs of geriatric patients while maintaining high-quality standards in healthcare. [8,26].

#### 3.1.5. Technical and Procedural Competencies

Technical and Procedural Competencies are indispensable in the provision of direct patient care in geriatric settings. Tasks such as giving injections and monitoring vital signs are key aspects of their role [25]. Critical technical competencies include the handling of medical devices, including urine catheters and ostomies; safe ambulation procedures; and the management of respiratory problems [23,25,31]. Evaluating and addressing fall-related hazards holds significant importance in the elderly care domain, as it underscores nurses’ responsibility to ensure safety and avert accidents [24].

#### 3.1.6. Quality Improvement and Evidence-Based Practice

Engagement in quality improvement initiatives and the application of evidence-based practice are essential for advancing geriatric care. The literature stresses the need for reflective practice and participation in research activities to develop one’s own clinical competencies and improve patient outcomes [33]. Nurses are encouraged to implement evidence-based guidelines in their practice and to monitor and evaluate the effectiveness of interventions to ensure continuous quality improvement [21]. Evidence-based practices include working with teams from diverse disciplines to bridge the gap between research and practice and analyzing data to reveal opportunities to further enhance the quality of care [14,24]. Ongoing professional development, particularly in areas related to evidence-based practice, is crucial for the continuous enhancement of nursing competencies.

## 4. Discussion

The findings of this scoping review provide a comprehensive overview of the core competencies essential for primary care nurses involved in CGAs. By identifying six key domains—Clinical Assessment and Diagnostic Competencies, Care Planning and Coordination, Professional and Interpersonal Competencies, Environmental and Systemic Competencies, Technical and Procedural Competencies, and Quality Improvement and Evidence-Based Practice—this review highlights the nursing competencies and skills required to provide person-centered care to the geriatric population in community settings.

In the Clinical Assessment and Diagnostic Competencies domain, core competencies are focused on accurately identifying the multifaceted health issues that older adults often face. Nurses are required to carry out comprehensive health assessments, including frailty, physical, cognitive, functional, nutritional and psychological assessments. According to the American Geriatrics Society (2016), thorough geriatric assessment by nurses is essential for detecting subtle health declines, such as frailty and cognitive impairments, which are often overlooked in routine care [36]. Furthermore, the study by Mowbray et al. (2023) demonstrated a positive correlation between frailty assessments and the identification of the need for CGAs by trained nurses, confirming the necessity for specialized interventions and thorough assessment [37]. Managing chronic conditions, such as multimorbidity and polypharmacy, is essential, with nurses also responsible for ordering and interpreting tests. Ellis et al. (2011) reported that CGAs significantly increase the likelihood of older patients being alive and living independently after emergency hospital admissions and emphasized that nurses play a key role in implementing CGAs [38].

The Care Planning and Coordination domain is a key point in creating the foundation of integrated geriatric care. The development of individualized care plans that reflect the individual health status, preferences, and values of older patients is an important skill for nurses, as it involves sharing decision-making with patients and families and ensuring the autonomy of patients. According to Chadborn (2019), the CGA has three main components: a structured assessment; the development of a care plan that works towards patient-centered goals; and improving patient satisfaction, prescribing practices, healthcare resource use, and quality of care [39]. Effective care coordination among healthcare providers is critical for maintaining continuity and preventing gaps in care. Aggarwal et al. (2023) reported that access to health services, systems, and policies was one of the most common concerns among patients receiving CGAs [40]. Additionally, Detering et al. (2010) emphasized the importance of advance care planning in improving end-of-life outcomes by ensuring that patient preferences, including cultural and spiritual considerations, are respected [41].

Establishing trust-based relationships with older adults and their families is essential, particularly through effective communication, empathy, and cultural competence. A meta-analysis by Cho et al. (2023) revealed that communication technology-based nonpharmacological interventions significantly reduce the behavioral and psychological symptoms of dementia in older adults [42]. Carpiac-Claver and Levy-Storms (2007) emphasized that effective communication between nurse aides and older adults significantly improves care quality in long-term care settings [43]. An important skill of nurses is the management of family dynamics and the inclusion of caregivers in the care process. Effective management of family dynamics and inclusion of caregivers in the caregiving process are also important nursing skills. In their study, Tate et al. (2024) emphasized that the relationship between effective communication, empathy, and cultural competence allows nurses to better understand the needs of elderly people and to ensure that care is provided in a way that respects cultural, spiritual, and social diverse backgrounds [10].

The domain of Environmental and Systemic Competencies in nursing practice, beyond patient care, includes systemic issues such as resource allocation, leadership, and advocacy for adequate staffing and equipment. Effective management of care environments, whether in outpatient, home-care, or long-term care settings, is essential to safe and collaborative care. Wong et al. (2013) reported on the role of nursing leadership in improving patient outcomes through effective resource management and team leadership, particularly in residential nursing settings where the nurse acts autonomously [44]. Similarly, Halcomb et al. (2017) emphasized the importance of autonomy and responsibility in primary care, which parallels the findings of this study [45]. Multidisciplinary collaboration forms the basis of the CGA, with nurses often serving as key coordinators. Leadership in managing care teams and contributing to practice development is vital, particularly in primary care settings, where nurses often work independently or in small teams.

Technical and Procedural Competencies for the CGA include performing routine clinical tasks such as administering medications, providing wound care, and using medical devices. Latimer et al. (2014) investigated nurses’ technical competence in wound care and effectiveness in reducing pressure ulcers in elderly patients [46]. Our study extends these findings by highlighting the need for specialized knowledge of age-related physiological changes, such as mobility limitations and skin fragility, that further impact wound care and overall patient safety. Nurses carrying out a CGA need to be able to handle medical equipment such as urinary catheters and ostomy tubes. In addition, nurses must have the technical expertise to monitor chronic conditions, assess risk factors such as fall risk, and manage emergencies such as delirium or respiratory distress. In Wang et al.’s study, geriatric-trained nurses appeared to have significantly greater levels of urinary catheter and stoma skills, and those able to assess and mitigate fall risk were better equipped to provide safe and effective care for elderly patients [47].

To improve quality and evidence-based practices in CGAs, nurses are expected to use evidence-based guidelines and participate in research to improve care outcomes. The scoping review revealed that nurses need the skills to assess the effectiveness of care interventions, monitor patient outcomes, and adjust practices on the basis of emerging evidence. This precautionary approach is in line with the findings of the study by Sorich et al. (2022), which highlights the importance of using Nurses Improving Care for Health System Elderly (NICHE) best care practices in creating geriatric programs. Furthermore, he emphasized that when nurses are equipped with the necessary tools and training in evidence-based practice, they are better able to contribute to quality improvement, thus leading to better patient outcomes and overall system efficiency [48].

The utilization of standardized assessment tools is imperative for consistency, accuracy, and efficacy in CGA. Instruments such as the Frailty Index and Clinical Frailty Scale facilitate the identification of older adults at risk for adverse health outcomes, while the Mini-Mental State Examination and Montreal Cognitive Assessment aid in cognitive screening [49]. Functional assessments, including the Barthel Index and mobility tests such as the Timed Up and Go test, provide insights into an individual’s capacity to perform activities of daily living and fall risk. The Geriatric Depression Scale is essential for detecting psychological distress among older adults. The integration of these validated tools into practice ensures a more comprehensive, evidence-based approach to CGAs, thereby enhancing care quality and patient outcomes [50].

Artificial intelligence (AI) is emerging as a transformative tool to augment CGA effectiveness. AI-driven analytics can assist nurses in detecting early signs of frailty, cognitive decline, and multimorbidity through the analysis of electronic health records and real-time patient data [51]. Machine learning algorithms can process gait patterns and mobility data to predict fall risks, enabling timely preventive interventions. Natural language processing tools can extract relevant clinical insights from patient records, reducing documentation burdens and enhancing efficiency. The integration of AI into CGA processes has the potential to refine assessment accuracy, improve care planning, and support more proactive, data-driven decision-making [52]. However, the implementation of AI in primary care settings necessitates further research to ensure its reliability, ethical application, and accessibility across different healthcare environments.

The importance of CGAs in the care of older adults cannot be overstated. As global aging continues to present significant challenges to healthcare systems, the CGA has emerged as a vital tool in managing the complex needs of this demographic. The role of nurses, particularly in primary care, is indispensable in the successful implementation of CGAs, given their close interactions with patients and their ability to deliver ongoing management and coordination of care.

However, the review also highlighted a significant gap in the standardization of CGA practices across Europe. Unlike other regions, there is no universally agreed-upon approach or set of tools for conducting CGAs [4]. This inconsistency creates challenges for nurses, who require comprehensive training and support to effectively utilize CGA tools in their practice. The lack of consensus on the roles and responsibilities of healthcare professionals in conducting CGAs, particularly in primary care, further underscores the need for clearer guidelines and educational interventions.

## 5. Limitations

Despite the valuable insights gained from this review, several limitations must be acknowledged. First, the geographical scope of the included studies was limited to Europe, which may reduce the generalizability of the findings to other regions. Furthermore, the focus on primary care nurses means that the competencies identified may not be fully applicable to nurses in other care settings, such as hospitals or specialist geriatric units. Future research should aim to broaden the geographic scope and investigate whether these competencies are transferable to different care settings, as well as focus on intervention studies evaluating the effectiveness of targeted training programs designed to improve CGA competencies.

## 6. Conclusions

This scoping review highlights the range of competencies required by primary care nurses to conduct CGAs effectively. By identifying six core competency areas, this review provides a framework that can inform both clinical practice and the development of training programs aimed at enhancing geriatric care. Addressing the current gaps in CGA practices and education is a pillar of ensuring that healthcare systems are equipped to meet the complex needs of an aging population.

## Figures and Tables

**Table 1:** Population, concept, and context (PCC) framework and search terms.

PCC Element		Search Terms
**Population**	Primary care nurses	Primary care nurses OR nurse practitioners OR registered nurses OR licensed practical nurses OR nurse clinicians OR gerontological nurses OR community health nurses OR family nurse practitioners
		**AND**
**Concept (Intervention)**	Core competencies, education, knowledge curriculum and skills in comprehensive geriatric assessment	Comprehensive geriatric assessment OR geriatric assessment OR geriatric functional assessment OR elderly assessment OR geriatric nursing OR nursing competencies OR nursing skills OR nursing education OR nursing training OR nursing practice OR professional development OR continuing education OR evidence-based practice OR clinical competence OR skill development OR technical expertise
		**AND**
**Context**	Comprehensive geriatric assessment in primary care settings	Primary care settings OR geriatric care settings OR outpatient care OR community health centers OR home health care OR long-term care facilities

**Table 2:** Overview of included studies.

Author (Year)	Title	Country	Framework/Aim Study	Study Design	Sample	Settings
Lyndon et al. (2022) [19]	Designing a nurse-led assessment and care planning intervention to support frail older people in primary care: An e-Delphi study	UK	To identify and establish expert consensus on important and feasible components of a nurse-led, comprehensive geriatric assessment (CGA)-based intervention for community-dwelling older people who live with frailty	e-Delphi study	33 primary and community specialist care nurses	Primary care center
Shen et al. (2024) [20]	Application, knowledge, and training needs regarding comprehensive geriatric assessment among geriatric practitioners in healthcare institutions: a cross-sectional study	China	To investigate the actual application, knowledge, and training needs of comprehensive geriatric assessment (CGA) among geriatric practitioners in China	Cross-sectional study	225 geriatric practitioners	Hospital
Jeffs et al. (2017) [21]	Identifying effective nurse-led care transition interventions for older adults with complex needs using a structured expert panel	Canada	A structured expert panel was established with the purpose of identifying effective nurse-led care transition interventions.	e-Delphi study	23 clinicians	Community-based home care, long-term care, acute care
Goldberg (2016) [22]	Development of a curriculum for advanced nurse practitioners working with older people with frailty in the acute hospital through a modified Delphi process	UK	To establish an expert consensus on the role description and essential competencies for ANPs working with older people with frailty to develop a curriculum.	e-Delphi study	34 registered clinician experts	NA
Lyndon et al. (2023) [14]	A nurse-led comprehensive geriatric assessment intervention in primary care: A feasibility cluster randomized controlled trial	UK	To determine the feasibility of a nurse-led, primary care-based, comprehensive geriatric assessment (CGA) intervention.	RCT	56 older adults with frailty	Primary care center
Safari et al. (2023) [23]	Comprehensive geriatric assessment delivered by advanced nursing practitioners within primary care setting: a mixed-methods pilot feasibility randomised controlled trial	UK	To explore the feasibility of identifying older adults with frailty and assess the subsequent implementation of a tailored CGA with care and support plan by Advanced Nursing Practitioners (ANPs).	RCT	160 people with frailty assessment by ANP	General practice center
Arrogante et al. (2023) [24]	Great geriatric syndromes: Acquisition of nursing competencies and undergraduate nursing students’ perceptions through high-fidelity simulation training	Spain	To evaluate the acquisition of the necessary nursing competencies for adequate management of great geriatric syndromes through high-fidelity simulation training and to explore undergraduate nursing students’ perceptions about this training.	Mixed-methods study	80 undergraduate nursing students	University
Tohmola et al. (2022) [8]	Competencies relevant for gerontological nursing: Focus-group interviews with professionals in the nursing of older people	Finland	To describe competence areas relevant in gerontological nursing.	Qualitative Study	27 gerontological nursing professionals	Healthcare organizations, university
Bing-Jonsson et al. (2016) [25]	Sufficient competence in community elderly care? Results from a competence measurement of nursing staff	Norway	Investigates the sufficiency of nursing staff competence in community elderly care.	Cross-sectional study	1016 nursing staff	Nursing home, home delivery services
Carlson et al. (2014) [26]	Registered nurses’ perceptions of their professional work in nursing homes and home-based care: a focus group study	Sweden	To illuminate how nurses working in nursing homes and home-based care perceived their professional work.	Qualitative study	30 registered nurses	Nursing home, home-based care
Piirainen et al. (2021) [27]	Challenging situations and competence of nursing staff in nursing homes for older people with dementia	Finland	To determine the prevalence of challenging situations in nursing homes of older people with dementia, characterize the nursing staff’s responses to such situations, and contribute to a model outlining competencies that dementia care nurses require.	Cross-sectional study	106 nursing staff	Nursing home
Dijkman et al. (2022) [28]	Developing a competence framework for gerontological nursing in China: a two-phase research design including a needs analysis and verification study	China	To identify and verify competencies for gerontological nurses that are needed to provide nursing care for the growing number of older people in all care settings.	e-Delphi study	NA	NA
Dudley et al. (2022) [29]	Addressing cultural competency and primary palliative care needs in community health nursing education	USA	To meet the primary palliative care needs of older adults, especially the underserved and those of color.	Qualitative study	34 nursing students	Community
Pakkonen et al. (2023) [30]	Effectiveness of an educational intervention to increase professional nurses’ person-centred care competence in long-term care of older people—Quasi-experimental study	Iran	Defining the components of emotional competence in caring for older people.	Qualitative study	25 participants (9 nurses, 12 nurse managers, 4 clinical instructors)	Long-term care
Kiljunen et al. (2019) [31]	Older people nursing in care homes: An examination of nursing professionals’ self-assessed competence and its predictors	Finland	To explore care home nursing professionals’ self-rated competence in older-people nursing and to identify predictors of this competence.	Cross-sectional study	781 registered nurses	Home-based care
Vatnøy et al. (2019) [32]	Exploring nursing competence to care for older patients in municipal in-patient acute care: A qualitative study	Norway	To examine the association between the integrated care competencies and cross-cultural competence of registered nurses.	Qualitative study	8 nurses, 2 physicians	Municipal in-patient acute care units
Stanyon et al. (2017) [33]	The competencies of Registered Nurses working in care homes: a modified Delphi study	UK	To define core competencies for RNs working in UK care homes.	e-Delphi study	NA	Home-based care
Kajander-Unkuri et al. (2022) [34]	Effectiveness of a combined web-based and simulation-based continuing education on home-care professionals’ competence to evaluate older people’s acute care needs in Finland	Finland	To investigate the effectiveness of combined web-based and simulation-based continuing education on home-care professionals’ competence regarding evaluating older people’s needs for acute care.	Quasi-experimental pre-test post-test study	254 home-care professionals	Home-based care
Burhenn et al. (2016) [35]	Geriatric assessment in daily oncology practice for nurses and allied health care professionals: Opinion paper of the Nursing and Allied Health Interest Group of the International Society of Geriatric Oncology (SIOG)	Switzerland	To outline the key domains of the GA and QoL for nurses and allied healthcare professionals when caring for older patients with cancer.	Opinion paper	NA	NA

## Data Availability

The datasets generated and/or analyzed during the current study are available from the corresponding author upon reasonable request.

## Supplementary Materials

The following supporting information can be downloaded at: https://www.mdpi.com/article/10.3390/geriatrics10020048/s1. Table S1: Quality Assessment of Included studies.
