# Supplementary material for: Comprehensive Geriatric Health Assessment Core Competencies and Skills for Primary Care Nurses: A Scoping Review
Source: Geriatrics (Basel). 2025 Mar 18;10(2):48. doi: 10.3390/geriatrics10020048 (PMC11932249; doi:10.3390/geriatrics10020048)
Supplement: Supplementary file 1 [file geriatrics-10-00048-s001.zip › geriatrics-3509533-supplementary.pdf]

Supplementary Material Table S1: Quality Assessment of Included studies.

Author , Year	Screening		Qualitative Studies					Quantitative randomized controlled trials					Quantitative non-randomized studies					Mixed Methods Studies					Score	Overall Quality
	**S1**	**S2**	**1.1**	**1.2**	**1.3**	**1.4**	**1.5**	**2.1**	**2.2**	**2.3**	**2.4**	**2.5**	**3.1**	**3.2**	**3.3**	**3.4**	**3.5**	**5.1**	**5.2**	**5.3**	**5.4**	**5.5**		
Arrogante (2023)	Yes	Yes																Yes	Yes	No	Yes	Yes	4/5	High
Bing-Jonsson (2016)	Yes	Yes											Yes	No	No	Yes	Yes						3/5	Moderate
Burhenn (2016)	Yes	Yes																Yes	Yes	No	Yes	Yes	4/5	High
Carlson (2014)	Yes	Yes	No	Yes	Yes	Yes	Yes																4/5	High
Dijkman (2022)	Yes	Yes											Yes	Yes	Yes	Yes	Yes						5/5	High
Dudley (2022)	Yes	Yes	No	Yes	Yes	Yes	No																3/5	Moderate
Goldberg (2016)	Yes	Yes																Yes	Yes	Yes	Yes	Yes	5/5	High
Jeffs (2017)	Yes	Yes																Yes	Yes	No	Yes	No	3/5	Moderate
Kajander-Unkuri (2021)	Yes	Yes											Yes	Yes	Yes	Yes	Yes						5/5	High
Kiljunen (2014)	Yes	Yes											Yes	No	Yes	Yes	Yes						4/5	High
Lyndon (2022)	Yes	Yes																Yes	Yes	Yes	Yes	Yes	5/5	High
Lyndon (2023)	Yes	Yes						Yes	Yes	Yes	Yes	Yes											5/5	High
Pakkonen (2023)	Yes	Yes											Yes	Yes	Yes	Yes	Yes						5/5	High
Piirainen (2021)	Yes	Yes											Yes	Yes	Yes	Yes	Yes						5/5	High

Safari (2023)	Yes	Yes						No	Yes	No	Yes	Yes											3/5	Moderate
Shen (2024)	Yes	Yes											Yes	No	Yes	Yes	Yes						4/5	High
Stanyon (2017)	Yes	Yes																Yes	Yes	Yes	No	No	3/5	Moderate
Tohmola (2022)	Yes	Yes	Yes	Yes	Yes	Yes	Yes																5/5	High
Vatnøy (2019)	Yes	Yes	Yes	Yes	Yes	Yes	Yes																5/5	High

Footnote: Mixed Methods Appraisal Tool (MMAT), version 2018, S1. Are there clear research questions? S2. Do the collected data allow to address the research questions?. Qualitative: 1.1. Is the qualitative approach appropriate to answer the research question? 1.2. Are the qualitative data collection methods adequate to address the research question? 1.3. Are the findings adequately derived from the data? 1.4. Is the interpretation of results sufficiently substantiated by data? 1.5. Is there coherence between qualitative data sources, collection, analysis and interpretation?, Quantitative randomized controlled trials: 2.1. Is randomization appropriately performed? 2.2. Are the groups comparable at baseline? 2.3. Are there complete outcome data? 2.4. Are outcome assessors blinded to the intervention provided? 2.5 Did the participants adhere to the assigned intervention?. Quantitative non randomized: 3.1. Are the participants representative of the target population? 3.2. Are measurements appropriate regarding both the outcome and intervention (or exposure)? 3.3. Are there complete outcome data? 3.4. Are the confounders accounted for in the design and analysis? 3.5. During the study period, is the intervention administered (or exposure occurred) as intended?. Mixed methods: 5.1. Is there an adequate rationale for using a mixed methods design to address the research question? 5.2. Are the different components of the study effectively integrated to answer the research question? 5.3. Are the outputs of the integration of qualitative and quantitative components adequately interpreted? 5.4. Are divergences and inconsistencies between quantitative and qualitative results adequately addressed? 5.5. Do the different components of the study adhere to the quality criteria of each tradition of the methods involved?
